# ‘Living in parallel worlds’ – bereaved parents’ experience of family life when a parent with dependent children is at end of life from cancer: A qualitative study

**DOI:** 10.1177/02692163211001719

**Published:** 2021-03-26

**Authors:** Cherith J Semple, Eilís McCaughan, Esther R Beck, Jeffrey R Hanna

**Affiliations:** 1 School of Nursing, Ulster University, Newtownabbey, Co. Antrim, UK; 2 South Eastern Health and Social Care Trust, Cancer Services, Ulster Hospital, Belfast, UK; 3 School of Nursing, Ulster University, Coleraine, Co. L’Derry, UK

**Keywords:** Parents, cancer, qualitative research, end of life, children

## Abstract

**Background:**

When a parent of dependent children (<18 years old) is at end of life from cancer, this has a profound impact on the family. Children less prepared for the death of a parent are more susceptive to poorer psychosocial adjustment in later life. There is a lack of understanding from the literature surrounding what support parents require, and how they navigate this end of life experience.

**Aim:**

To explore bereaved parents’ experience and needs for families when a parent is at end of life from cancer with dependent children.

**Design:**

In-depth, semi-structured qualitative interviews were conducted with 21 bereaved mothers and fathers, identified from the general public, a family support service and hospice. Data were analysed thematically.

**Results:**

Parents often live in ‘parallel worlds’ throughout the end of life period. In one world, ‘living in the moment’, cherishing the ordinariness of family life, remaining hopeful treatment will prolong life, whilst adapting as the illness unfolds. The other world presents as ‘intermitted glimpses that death is approaching’, shadowed with painful emotional concerns surrounding their children and the future. At the end, death rapidly approaches, characterised as suddenly ‘falling off the cliff’; placing significant demands on the well-parent.

**Conclusions:**

Amidst challenges, clinicians should provide parents with clear information surrounding a poor prognosis, so families can plan and prepare for parental death. There is a need for healthcare professionals to engage, encourage and equip parents, as they prepare their children throughout the end of life experience for the inevitable death of a parent.

**What is known about the topic?**

• End of life is an especially stressful and disruptive experience for families to navigate with dependent children (<18 years old).
• Parents of dependent children often feel ill-equipped and uncertain how best to prepare and support their dependent children for the impending parental death.
• Parents’ desire and need psychological support and guidance from health and social care professionals but in reality, this is often lacking.

**What this study adds?**

• Parents are living in ‘parallel worlds’ throughout most of the end of life period; mainly characterised by making the most of everyday ordinariness in an attempt to maintain a sense of normality and provide stability for the children, with only intermittent glimpses into the other painful world that parental death is approaching.
• Lack of clear, realistic prognostic information; coupled with hope of life-extension from evolving treatments contributes to a lack of advanced planning and preparing children for parental death.
• Postponement of advanced planning by parents is impacted by emotional distress and pain to confront the grim reality of impending death; reconciling that such preparations will occur nearer to the time of death, but more often never taking place.

**Implications for practice, theory and/or policy**

• Clinicians should provide parents with clear and realistic information surrounding their advanced cancer, with honest disclosure on prognostic estimates from available treatments.
• Family-centred communication should be seen as a process, with health and social care professionals encouraging and equipping parents to start the difficult conversations soon after receiving the poor prognosis, to avoid crisis management when the ill-parent is actively dying or throughout the immediate bereavement period.
• To facilitate a better end of life and bereavement experience for the whole family, parents should be encouraged to create memories for the future by capturing life as it naturally happens.

## Introduction

It is estimated that 20% of patients of a parenting age with cancer, experience death whilst parenting dependent children (<18 years, hereafter referred to as children).^1^ The end of life trajectory for families is highly disruptive and unpredictable.^2^ For the purpose of this study, end of life is when a person is expected to die from cancer within twelve months.^3^ Challenges that are likely to confront parents throughout this period include a reduced availability for parenting; changes to parental roles; heightened distress as they prepare children for parental death; and, or financial implications.^4,5^

One of the intricate and complex challenges faced by parents is how to best prepare and support their children throughout this end of life experience.^5^ Children with an understanding of the dying process and are prepared for the death of their parent show quicker adjustment than those not informed.^6^ Conversely, children lacking support and involvement are more susceptive to psychological difficulties throughout childhood, including a decline in education and increased levels of guilt and mistrust.^7,8^ These negative effects can be prolonged, with heightened susceptibility to substance abuse and mental health issues in later life.^9^ There are clear benefits of open communication at end of life, in that it maintains and sustains the parent-child relationship, mediating for such adversities.^7^

A recent systematic review highlighted parental obstacles in preparing children for the death of a parent.^4^ These included parents (both ill and well-parents) lack of readiness to accept the ill-parent’s declining health and, or hope for life-extending treatments. While parents often require supportive guidance from professionals,^4^ such family-centred care is often inadequate in routine clinical practice.^5,10^ To better understand how and when family-centred cancer care can be best facilitated and provided, there is a need to gain an improved understanding of parents’ needs and how they managed the end of life experience with their children.

### Aims and objectives

Through interviewing bereaved parents, this study aims to explore the experience and needs of parents with dependent children, when their mum or dad is at end of life from cancer. Through the lens of bereaved parents, the objectives of the study are to explore how parents:

1. perceived they managed family life,
2. communicated with their children,
3. prepared their children for the death of mum or dad,
4. could be best supported as they managed family life.

## Methods

A qualitative design using semi-structured interviews was employed. The study is reported following the Consolidated Criteria for Reporting Qualitative Research guidelines.^11^

### Participants

Twenty-one parents were recruited between April 2018 and February 2020. Convenience sampling identified participants from the general public, a family support service and hospice throughout Northern Ireland (NI), UK. The family support manager and social worker at respective organisations identified eligible participants (Table 1) from their databases. They were directly mailed study details, with no further introduction, explanation or follow-up. A total of 18 out of 35 parents did not respond to the study invitation.

**Table 1.** Participant eligibility criteria for inclusion to the study.

1. Experienced the death of a co-parent to cancer.
NB: No limits were applied surrounding minimum period between death and inclusion to the study.
Rationale: How an individual is coping in their grief is subjective and the emotions an individual experiences at three months post-death may be similar to that of 9 years post-death.^12^
Upper-limits were up to 5 years following the death for those parents identified from the family support service and hospice. These were in line with both sites compliance to storage and handling of the parent’s personal information.
No lower or upper-limits were applied to parents who volunteered to participate in response to the study’s public advertisement.
2. Dependent children (<18 years old).
3. Resided in Northern Ireland.
4. Ability to speak and understand English.

Volunteer sampling techniques were used to assist accrual and representation of hard-to-reach families beyond support groups and hospice services.^4^ A public advert was developed by the research team and study’s expert group; published on three occasions in a national newspaper and displayed in 183 public spaces (clinical settings, leisure and community centres and supermarkets in NI). Four interested parents confirmed their inclusion to the study with the fourth author [J.R.H.]. Written consent was obtained at the time of interview.

### Data collection

A topic guide with open-ended questions was developed; guided by the literature alongside the research and expert group; iteratively modified enabling follow-up in subsequent interviews (Table 2). Interviews were completed when no further categories were identified. Interviews were conducted face-to-face by the first author [C.J.S], who was an experienced qualitative researcher and senior cancer nurse specialist, and the fourth author [J.R.H] who was an academic researcher; neither of whom had prior relationships with the participants. In-depth, semi-structured interviews were audio-recorded, lasting between 60 and 120 min at a venue convenient for participants [home (*n*16), work (*n*2), support service site (*n*3)].

**Table 2.** Semi-structured topic guide used to guide the conduct of the study.

Initial topics based on the literature, research aim and objectives and expert group.
1. Exploration of family life between receiving the incurable diagnosis until the parent’s death.
2. Communication between parents and children from receiving the incurable diagnosis, until the parent’s death.
3. Parent’s emotional readiness to share the incurable diagnosis with the children.
4. Exploration surrounding how parents prepared their dependent children for the death of mum or dad.
5. Emotional, practical and other (unmet) needs throughout the experience from receiving the incurable diagnosis until the death.
Sample of additional topics following identification of initial categories.
1. Pre-existing parental roles in sharing information with the children surrounding mum or dad’s incurable diagnosis and declining health.
2. Exploration of parent’s engagement (or lack of) in memory activities.
3. Navigating parental responsibilities and extended support network when the ill-parent was imminently dying.
4. Forward planning for the future

### Data analysis

Audio-recordings were transcribed verbatim and managed using NVivo v.12; facilitating analysis using Braun and Clarke’s thematic analysis framework.^13^ To ensure rigour, credibility and trustworthiness, all authors read the transcripts. However, the first author [C.J.S] and fourth author [J.R.H] read and reread the transcripts to gain a sense of each parent’s story, then independently coded the data, detailing inductive descriptive codes, using the participants’ own language when possible. Through an iterative process of reviewing and discussing data, themes were identified and refined at research team meetings [C.J.S, E.McC, E.R.B, J.R.H].

### Ethical considerations

Parents opted-in to the research, and were made aware of their right to withdraw and this would not impact current or future care from the hospice or family support service. A distress protocol was established, and a support pack provided to parents as part of the study’s debrief. Data protection procedures were observed and assurances of confidentiality were given. Ethical approvals were obtained at institutional and national levels [REC:17/SW/0155].

## Results

Twenty-one bereaved parents were recruited, totalling 12 mothers and nine fathers. Parents were identified from a family support service *(n14)*, a hospice *(n3)* and a public advertisement *(n4).* Participants were between 5 weeks and 6 years after the death of the parent *(mAverage = 14.6 months)*, but mostly (*n15)* less than eighteen months from the death of the parent. Sample characteristics are reported in Table 3.

**Table 3.** Sample characteristics of the parents included in the study.

Variable	*N*
Participant (parent)	
Father	9
Mother	12
Cancer site	
Pancreatic	2
Lung	1
Bowel	1
Breast	2
Head and neck	1
Glioblastoma	3
Melanoma	2
Renal	1
Esophageal	3
Angiosarcoma	1
Liver	2
Ovarian	2
Marital status at time of death	
Married	20
Partner	1
Gender/age of children	
Boy, 0–11 years old	15
Boy, 12–18 years old	7
Girl, 0–11 years old	19
Girl, 12–18 years old	12
Recruitment source	
Hospice service	3
Public advert	4
Family support service	14
Place of death	
Home	5
Hospice	7
Hospital	9
Socioeconomic details	
Highest level of completed education	
Secondary level (GCSE/A-level)	6
Skills based qualification	2
Bachelor’s degree	11
Master’s degree	2
Employment status at time of death	
Full-time work	5
Part-time work	2
Leave of absence	10
Maternity leave	1
Unemployed	3
Main source of household income at time of death	
Employment	7
Statutory sick pay	9
Statutory maternity pay	1
Social security funding	4

**Figure 1.**
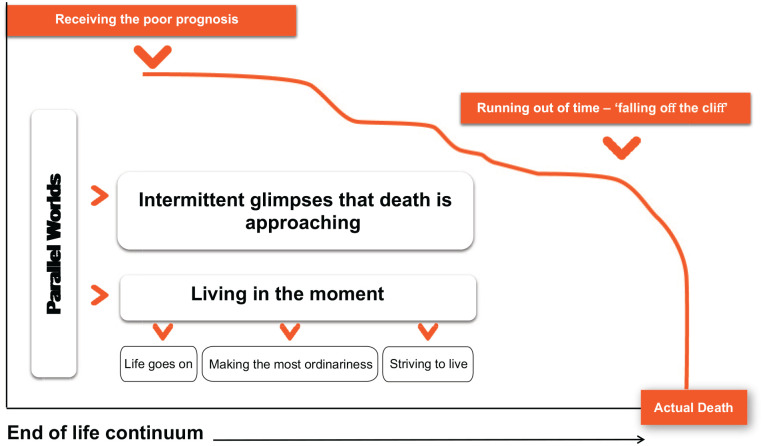
End of life continuum when a mum or dad with dependent children is dying from cancer.

For the majority of families, the ill-parent’s health gradually declined throughout most of the end of life experience, until a point in time when their body became less responsive to treatment; evidenced by rapid signs of physical deterioration. In contrast, for a few families, the illness trajectory was limited to a period of weeks or days from accessing healthcare to parental death. The data below are representative of the longer disease trajectory. Figure 1 constructs this continuum, highlighting some of the key interconnected complexities. Eight sub-themes were identified, categorised into five broad themes: (1) emotional readiness to share the poor prognosis with the children, (2) parallel worlds – living in the moment and intermittent glimpses that death is approaching, (3) striving to live – hope from treatment and spiritual faith, (4) running out of time – falling off the cliff and (5) how best to support parents across the end of life continuum.

### Theme 1: Emotional readiness to share the poor prognosis with the children

As reported by the bereaved parent, both the ill and well-parents (hereafter referred to as parents when referring to both) most distressing concern when diagnosed with a poor prognosis was for their children. Most parents highlighted that initially it was too painful to consider telling the children mum or dad’s cancer had returned. There was an evident ‘need to get their head around it first’ before sharing with others, never mind the children. Barriers precipitating delay in disclosure was parents’ belief their children should not be told until their parent was ‘evidently deteriorating’. They perceived this was protecting and shielding children from prolonged pain and upset. In other circumstances, parents felt it was best to wait until key events or celebrations had passed, such as school exams, birthdays and Christmas. On occasions, there appeared to be tension between the parents relating to appropriate timing of sharing key information. More often, there was a greater sense of urgency from the well-parent to inform children that the cancer was advanced and progressive.

*“I started to get frightened. The cancer was spreading at a pace faster than we anticipated. But he was just Mr. Positive, whereas I suddenly was panicking and thinking I’m going to be left here on my own with three kids.” [parent interview 17]*

Generally, the well-parent wanted to be involved in communicating this significant news. Their reasoning was twofold: to protect and minimise their spouse’s distress, but cognisant they would have ongoing parenting responsibility, wanting to assess children’s reactions. There were other significant conversations to follow, which proved emotive as the cancer progressed and death was approaching. Of note, the timing of these conversations was predominately influenced by parents’ emotional readiness to share the news, not children’s developmental age.

“W*ent back on Monday morning and the cancer had spread to the brain. So that was another massive blow. You know you’re slipping down, but each slip is a massive blow.” [parent interview 20]*

Furthermore, well-parents had copious concerns that weren’t openly share with their spouse, spanning: how they were going to manage parenting responsibilities as the ill-parent declined; how the death of a mum or dad would psychologically impact the children; and fears surrounding raising the children on their own.

### Theme 2: Parallel worlds – living in the moment and intermittent glimpses that death is approaching

Many parents were living in parallel worlds throughout most of the end of life experience, characterised by mainly choosing to live in the moment. Parents’ savored everyday life, with only occasionally thinking about the impending death. Everyday life was encapsulated by the following sub-themes: (1) life goes on – adapting to the poor prognosis in the family, and (2) making the most of ordinariness.

#### Sub-theme 2.1: Life goes on – adapting to the poor prognosis in the family

Within the last year of life, it appeared important for ill-parents to maintain previous parenting responsibilities, despite physical constraints. This included helping with homework and making lunches, thus providing opportunities to ‘parent’. This was driven by an ardent effort to protect the children; creating a sense of normality and security. Also, parents wanted their children to continue with their usual activities such as going to school and spending time with friends, perceiving this lessened children’s emotional distress.

*“So she picked the kids up and taking them home again. She loved all that. That’s it being mummy. That’s all she ever wanted to be was a mummy. So as long as mummy was in the car, you know it’s fine. Mummy’s still there. To be fair, as long as mummy is still here, I can go and get a hug anytime I want and that’s good” [parent interview 02]*

Maintaining routine became more effortful and less achievable as the ill-parent’s health was declining, especially if hospitalisation was required. Nonetheless, immense resourcefulness was demonstrated to continue with some daily routines.

*“Bert said to me ‘tomorrow can I use the desk in your bedroom because I want to do some work’ and he said ‘this isn’t bad I’ll sit up here’. And he died the next day. Those weeks seemed to condense into hours” [parent interview 19]*

Often, as death was imminent the ill-parent continued to be ‘involved’ in everyday family life. Although doing less ‘practical’ parenting, their role remained significant such as listening to the child’s school day. Being in the same room as the child enabled a ‘parental’ presence, which provided connectedness with their child.

*“Sally [ill-parent] would have sat in her wee chair so they [children] could have sat watching TV while Sally was there. Maybe with her eyes closed sleeping or whatever, but Sally was still there, and in their [children] eyes involved” [parent interview 02]*

#### Sub-theme 2.2: Making the most of ordinariness

As parents anticipated future disruptions and losses, there was an evident keenness to do more family-related activities. When the ill-parent felt ‘well enough’, outings were ‘engineered’ to optimise these experiences.

*“If he was having a good day, I’d have said come on we’re going down to the Bay or we’d go here or go there” [parent interview 04]*

Once parents processed that death was hastily approaching, despite their fears and sadness, they intentionally focused on cherishing everyday happenings, such as going for a walk. Many more photographs and short videoclips were taken, motivated to ‘capture the moment’. Alongside this, celebrations such as birthdays and achievements were more consciously experienced, with parents ‘finding joy’ in these moments.

*“Once you get through that phase, he’s not going to do, I just wanted to make sure that Maurice [ill-parent] and Abbey [child] had memory things and the simplest wee things. You know he loved getting the lego out and him and her. And taking photographs without them looking. Just so she has things that are like daddy did that with me’ [parent interview 07]*

Some parents did write letters for the future, but often these memory activities commenced only as death was hastily approaching. Due to physical and cognitive cancer-related limitations, they were often ‘incomplete’ or ‘filled with mistakes’. In reality, most ill-parents had not ‘got around to doing them’, as it was too painful to ‘put in writing’ the certainty of not being around for the children’s future. Other parents felt the recommendation by professionals to produce memory boxes or related items, created a background pressure on them.

*“Chiara had written a list of things to do, but she didn’t get through them all. She had hoped to do memory boxes and things like that, but I think it was too painful for her to be quite honest. It was too emotionally difficult” [parent interview 14]*

For ill-parents who spent time in an inpatient palliative care unit, there was an appreciation when professionals facilitated the ‘capturing the moments’ and ‘parenting’ opportunities, such as going to a café or ‘party time’ in mum or dad’s room. Similar family-centred activities were not reported when parents were in the acute hospital setting.

*“In Marie Curie, we had party dates with the kids, or went out somewhere and went to the cinema or went out to a restaurant or café” [parent interview 10]*

### Theme 3: Striving to live – hope from treatment and spiritual faith

Although parents reported knowledge that mum or dad’s cancer was advanced with no curative treatment options, some remained hopeful of many more years to see their children grow-up. Parents’ pursed and were often offered a series of treatments; when one failed, another was considered. It appeared each treatment ushered in hope and optimism, especially novel immunotherapy treatments. Some parents placed excessive optimism in modern cancer care, maintaining hope right up to the final weeks and days of life. Often, it seemed to be the ill-parent who was placing more hope in effective, life-extension from treatment, with the well-parent more realistic that death was closer.

*“Dominique [ill-parent] would have said it’s okay we’re getting treatment and everything’s stable. And everyday we’re getting closer to them coming up with something new. He [ill-parent] thought he would beat this, so I couldn’t be negative” [parent interview 11]*

During end of life, a few parents had a strong religious or spiritual belief that the parent would be healed from cancer, and for some this thinking remained until their actual death. Such hope meant a lack of advanced planning. For other parents, there appeared to be a comfort derived from their spiritual faith, reporting no fear of dying and a hope of going to ‘a better place’ after their death.

*“Dave [ill-parent] had a really strong faith and so he didn’t really have a fear of death. He believed he would go to heaven. He wasn’t scared about that which I think really helped” [parent interview 21]*

### Theme 4: Running out of time – Falling off the cliff

For variable periods of time, it appeared parents coped by adjusting and getting through the ups and downs of treatments, with ‘home life going on as usual’. Then the ill-parent declined very quickly, moving speedily to the ‘dying period’; typified in days or short weeks. At this pivotal timepoint, parents did get a sense they were ‘running out of time’, describing the feeling like ‘falling off the cliff’.

*“Suddenly he nose-dived over Christmas. Boxing day, he made it out on Boxing day and he headed out with his cousin and went down to The Inn [local restaurant]. Came home and managed to sit up with everyone, but basically he nose-dived on a daily basis and died early January” [parent interview 05]*

Despite parents’ devoted focus on the needs of children throughout the end of life trajectory, when death became imminent, children appeared to be on the side-lines. There was an obvious lack of parenting capacity from either parent, as the well-parent’s attention was focused on the ‘dying partner’. Input from close social networks (mainly family members) was frequently necessary for the practical aspects of parenting. Some parents highlighted the challenge that hospitals, unlike the hospice, presented strict visiting periods that coincided with times of basic family routine such as evening mealtime and bedtimes. However, in the last few days when the parent was actively dying in the home or hospice, parents wanted ‘uninterrupted’ time with their close family unit and limited visits from ‘outsiders’.

### Theme 5: How best to support parents across the end of life continuum

Despite bereaved parents reporting a need for instructive support from professionals to aid preparations for parental death, such care was often lacking. This is discussed under two sub-themes: (1) honest information on prognosis and guidance required from health and social care professionals and (2) encouraging parents to forward plan for the future.

#### Sub-theme 5.1: Honest information on prognosis and guidance required from health and social care professionals

Most parents wanted to be honestly, sensitively and realistically informed that the cancer was incurable. There was a desire that prognostication conversations were professional-led (not having the parents asking), providing clarity regarding ‘time left’; especially when approaching the final weeks and days of life. During significant conversations, parents focused not only on the language used, but professionals’ emotional cues. On reflection, parents reported a hinting and ‘hiding behind’ phrases like ‘making the most of time left’ or ‘what’s important is quality of life’. A lack of clear prognostic disclosure meant some families feeling unprepared and ‘launch padded’ into the ‘active dying’ phase.

*“I could see Darryl [ill-parent] deteriorating. She [palliative care nurse] said we are talking days. That was like, gutted. I had no idea and he died on the Monday. . . I was thinking months, maybe two, three months” [parent interview 16]*

Providing information on prognostication appeared complex, augmented when tensions between parents was evident. Also, how this sensitive information was perceived and received was influenced by parents’ personal faith and hope in life-extending treatment, acknowledging the need for a skilled communicator.

It appeared most parents’ trudged through a period of living with advanced cancer, ‘nervously aware’ of the need to tell the children. Postponing disclosure until a rapid deterioration was apparent often lead to a ‘crisis’ point, enveloped with panic and an abundance of concerns. There was a poignant lack of supportive care from professionals regarding the children, with involvement mainly directed towards the physical needs of the ill-parent. On reflection, bereaved parents stated preparing their children for parental death was an ‘isolating’ experience. Primarily, parents wanted words and language on how to best share this distressing news and answer their children’s difficult questions.

*“What on earth am I going to tell the children. I needed guidance. What’s the right approach to tell the children. And I just got so frightened suddenly because the cancer had gone into the brain and it was in the bones” [parent interview 09]*

For those parents who ‘took on’ the emotive challenge of telling their children, mum or dad was eventually going to die, considered it a great relief once ‘it was out there’. In contrast, parents who reported not telling the children until after the death reflected that earlier, open and honest conversations may have been helpful.

*“I don’t think it would have been as hard to tell on the day (of the actual death) if I would have prepared her (daughter) you know, daddy hasn’t got too long left” [parent interview 15]*

#### Sub-theme 5.2: Encouraging parents to forward plan for the future

Proactively engaging with future plans such as financial matters at the commencement of the end of life period proved helpful, otherwise, these processes were cumbersome post-bereavement. Bereaved-parents advocated the importance and helpfulness of forward planning pre-bereavement, before the opportunity passes by. When possible, they should include managing practical matters such as finances, ‘wills’, passwords on accounts, personal elements such as funeral wishes and future aspirations for the well-parent and children. Whilst it was perceived that more personal and intimate preparations and conversations would happen ‘nearer the time of death’, in reality, most parents had limited meaningful conversations surrounding future family-life.

*“We really didn’t get into those conversations and I guess that is one of my regrets. But when someone is fighting so hard, how do you say ‘well what are we going to do when you die’. So the only thing I made sure was that we got her will signed off” [parent interview 13]*

## Discussion

Findings from our study highlighted parents were often living in parallel worlds throughout the end of life period, which appeared to impact their readiness to prepare their children for the impending death of the ill-parent. In one world, it was important for parents to adapt to the unfolding prognosis within the family, in their attempt to maintain some sense of normality. Stiving for normality was an endeavor to provide stability for the children.^14–16^ Akin to other studies, parents pinned hope that treatments may prolong life.^17–19^

In another world, parents were experiencing emotional turmoil, as they contemplated telling their children mum or dad was eventually going to die.^4^ An important factor impacting parents’ ability to prepare children for parental death was a lack of clear prognostic information. Presenting parents with prognostic information is vitally important to promote their understanding of the prognosis; inform treatment decision-making; and facilitate advance care planning, but is complicated today by availability of novel immunotherapies.^20,21^

Parents also felt ill-equipped, lacking appropriate language to use when having difficult parent-child conversations.^22^ Furthermore, some parents did not want to acknowledge and accept the reality that the ill-parent would die from the cancer. For those families, postponing disclosure led to ‘crisis management’ when the ill-parent was rapidly declining. When parents keenly wanted and needed instructional support, this was often lacking, with professional care directed towards the physical needs of the ill-parent. Similar findings have been reported by Hanna et al.^5^ and Franklin et al.^10^ To help reduce parenting tensions, supportive care should not be directed to the ill-parent alone, but both parents.^23^ However, professionals have reported this as an emotionally challenging aspect of their role, feeling ill-equipped, necessitating training to facilitate family-centred care in practice.^5,10^

Literature reports parents’ desire to spend quality time together as a family when mum or dad is dying from cancer, creating memories for the future.^4,24,25^ In this study, it was important for parents to capture family-life (mentally and tangibly with photographs and videoclips) as it was naturally happening. This was instead of creating elaborate plans such as going on foreign holidays.^26^ While literature suggests parents are often encouraged by professionals to engage in activities such as memory boxes,^27,28^ it was too emotionally difficult for parents. It may be suggested that encouraging parents to engage in memory activities creates an unnecessary pressure, and too intrusive at an emotionally difficult time.^29^ Rather, it may be helpful if parents were encouraged by professionals to make the most of ordinary everyday activities, taking and capturing each day as it comes, when the ill-parent still feels well enough to participate.^19^

During the parent’s final weeks of life, there was an essential need to have significant input from close family and social networks, necessitated by the ‘absence’ of the ill-parent and the well-parent.^30,31^It could also be purported that additional practical support was necessary due to fixed visiting periods in the acute setting, often coinciding with necessary family routines. There is a need for flexible visiting arrangements when a parent of dependent children is at end of life. Furthermore, as highlighted in the findings, parents’ desire for uninterrupted time when mum or dad is actively dying to facilitate quality family time together.^32^

It was suggested forward planning facilitated ease for the bereaved parent following the death. Similar findings have been reported within the bereavement literature.^33^ For other parents, it appeared postponement was impacted by finding it too painful to prepare for not being around for the children or spouse,^14,34,35^ and feeling such preparations would occur closer to the time of death.^20^ To facilitate a better bereavement experience, professionals should encourage parents to make necessary preparations and openly communicate about the future soon after receiving the poor prognosis;^36^ as death often occurs sooner than expected.

### Limitations and directions for future research

Bereaved parents included in this study are representative of those from two-parent or ‘significant adult’ families. It is unclear how single parents and those with complex family set-ups navigate this end of life experience, despite efforts to gain broader representation by public advert. The sample recruited participants within NI, being one of the four countries within the UK, viewed more culturally homogenous.^37^ Nonetheless, in recent years NI has become increasingly secular and ideologically diverse.^38^ The interviews were conducted retrospectively and bereaved parents’ perspectives may have been impacted by their experiences from the period following death. Longitudinal studies should be considered, following families (parents and children) throughout the end of life trajectory.

## Conclusion

This study highlighted the complex and highly emotive experience for parents of dependent children, when mum or dad was at end of life from cancer. There is a need for parents to be encouraged to make significant preparations shortly after receiving a poor prognosis. This includes informing the children of the parent’s poor prognosis and inevitable death, as well as managing practical and financial matters for the future. To help facilitate these preparations, health and social care professionals should be providing parents with clear prognostic information surrounding the reality of their poor prognosis, with necessary updates as death becomes imminent. Alongside this, professionals should be providing the ‘language’ for parents to engage with honest and age-appropriate conversations with their children, informing them that mum or dad is going to die.
